# Feasibility and content validity of the PERF-FIT test battery to assess movement skills, agility and power among children in low-resource settings

**DOI:** 10.1186/s12889-020-09236-w

**Published:** 2020-07-20

**Authors:** Bouwien C. M. Smits-Engelsman, Emmanuel Bonney, Jorge Lopes Cavalcante Neto, Dorothee L. Jelsma

**Affiliations:** 1 Department of Health & Rehabilitation Sciences, Faculty of Health Sciences, University of Cape Town, Cape Town, South Africa; 2 Institute of Child Development, University of Minnesota, Minneapolis, USA; 3 Department of Human Sciences, Bahia State University (UNEB), Jacobina, Brazil; 4 Department of Physiotherapy, Federal University of São Carlos (UFSCar), São Carlos, SP Brazil; 5 Developmental and Clinical Neuropsychology, University of Groningen, Groningen, the Netherlands

**Keywords:** Movement skills, Skill-related physical fitness, Physical fitness, Children, Low-resource settings

## Abstract

**Background:**

Numerous movement skills and physical fitness tests have been developed for children in high-income countries. However, adaptation of these tests to low-resource settings has been slow and norms are still unavailable for children living in low-income communities. The aim of this paper was to describe the development and validation of the Performance and Fitness (PERF-FIT) test battery, a new test to assess motor skill-related physical fitness in children in low-resource settings.

**Method:**

The PERF-FIT test was developed in a stepwise manner. This involved defining the relevant domains of the construct of interest and selecting and evaluating test items. The Content Validity Index (CVI) was used to estimate content validity. Following development of the PERF-FIT test, a preliminary study was performed to validate items and to examine the feasibility of implementing the test in a low-resource community. Structural validity was also determined based on data from eighty (*n* = 80) children (aged 7–12 years) using principal component analysis.

**Results:**

The CVI for the throw and catch item was 0.86 and 1.00 for the other nine items, leading to a total CVI score of 0.99. The hierarchical sequence of the item series was demonstrated by highly significant (*p* < 0.001) linear trends, confirming the increase in difficulty of subsequent items. Principal component analysis revealed three factors; the first component is represented by locomotor skills that require static and dynamic balance, the second component by throwing and catching items and the third component by agility and power items. These findings suggest that it is feasible to implement the PERF-FIT in low-resource settings.

**Conclusion:**

The PERF-FIT test battery is easy to administer and may be suitable for measuring skill-related physical fitness in in low-resource settings. It has excellent content validity and good structural validity. After minor adaptions, further studies should be conducted to establish normative values, evaluate reliability, and document criterion and cross-cultural validity of this test.

## Background

Physical fitness is a powerful health indicator in children and adolescents [1–3]. High levels of physical fitness are believed to normalize weight and improve mental health in children [4]. Proficient movement skills and optimal fitness provide strong basis for children to participate in everyday activities [5]. As a result, there is a global push for monitoring physical activity and fitness in children at the population level [2, 3, 6]. This call is supported by recent evidence suggesting drastic declines in physical fitness in children worldwide [7]. Despite the growing interest in children’s physical health [8], there seems to be limited data on movement skills and physical fitness among children in low-resource settings. Specifically, population-based data on movement skills and skill-related physical fitness are still lacking. The lack of population-based data on these variables may be attributed to limited accessibility to standardized movement skills and skill-related physical fitness assessments in many low-resource settings. Consequently, early detection of deficits in movement skills and skill-related physical fitness remains an enduring challenge. It is therefore necessary to identify new methods to assess movement skills and skill-related physical fitness in low-resource settings.

Numerous physical function or fitness tests have been developed for children and adolescents [9, 10]. The Eurofit, Alpha and Fitnessgram tests have gained much traction around the world [10–12]. Recently, the FITness testing in PREschool children was also developed for children in the young age group [13]. While these fitness tests are valid and reliable, most of them do not assess movement skills (e.g. throwing and catching, jumping, hopping, and balance), attributes known to be critical for promoting active lifestyles in children [14]. In addition, existing tests were developed in Western populations and norms are mostly based on children in high-income contexts. Few tests have been specifically designed to assess movement skills, agility and power tests or skill-related physical fitness in children in low-resource settings. Therefore, it is necessary to develop a test that takes into account societal challenges (e.g. the lack of funding to purchase standardized movement skill assessments developed in Western countries) in these areas. Importantly, such test should incorporate tasks that are familiar to children in low-socioeconomic backgrounds. Many children in these settings are less familiar and skilled in sport specific motor patterns because of the absence of sport facilities and unavailability of physical education curriculum in schools. Active transportation and active play tend to be more common in these communities. Therefore, contextual differences may cause children to respond or perform poorly on tests developed in Western societies. Some may even demonstrate “developmental delays” which may reflect the mere lack of appropriate instruction and/or limited practice of motor skills [15].

Although many valid and reliable motor performance tests exist [5, 10, 13, 16–23], there are several limitations that need to be acknowledged. These include (1) they lack adaptation to low-resource settings (2) they lack norms for children in these settings and (3) some are too expensive limiting uptake and utilization by people working in such environments (4) only few assessments have items that measure skill-related physical fitness. In addition, the use of task loading to increase item difficulty depending on the performance level is rarely used. Items are either the same for all age groups [as seen in Bruininks-Oseretsky Test of Motor Proficiency- second edition (BOT-2)] [17], which often yields ceiling effects, or use different items for different age bands [e.g. Movement Assessment Battery for children, second edition (MABC-2] [18], which makes it harder to compare children of different age groups or follow children longitudinally. The *Körperkoordinationstest für Kinder* (KTK) [20] uses the same items but increases task difficulty for two of the four items. In the hopping task, children start hopping over a certain height based on their age and depending on their performance the task difficulty is either increased or decreased. Also, on the balance beam items, children have to walk over beams that get smaller, which makes the task increasingly difficult. However, KTK only measures one aspect of motor performance namely dynamic balance [20]. The Canadian Agility and Movement Skill Assessment (CAMSA) [21] assesses fundamental, complex and combined skills in children aged 8–12 years and is suited for testing groups of children in an educational setting. However, this test does not have normative values for children in low-resource African or South American countries.

To address these limitations, we developed a new field-based test called the Performance and Fitness (PERF-FIT) test battery. The PERF-FIT test was developed for health professionals (physical and occupational therapists) and movement educators (physical education teachers and coaches) working in low-resource settings. This test combines movement skills, agility and power and incorporates progressive increase in task difficulty (i.e. task loading) to all skill items. The most commonly used movement performance tests such as the MABC-2 and BOT-2 require large initial costs (which is justified given the costs for standardization of materials e.g. the different balance beams used in both tests) and high ongoing costs associated with forms and equipment replacements. Being cognizant of the socio-economic challenges prevalent in low-resource settings, we elected to use materials that are relatively cheap and can be easily obtained in these areas. The PERF-FIT materials should be reasonably priced if made by users (approximately 50 US dollars). The testing equipment involved simple and cheap items such as tape measure, rectangular foam, and soda cans. These items are affordable and easily accessible in many low-resource settings. The aims of this paper are fourfold (1) to describe the development of the PERF-FIT, (2) to examine the content validity (3) to report on the feasibility of implementation and (4) to examine the structural validity using preliminary data from 7 to 12-year-old children living in Brazil.

## Methods

**Fig. 1:**
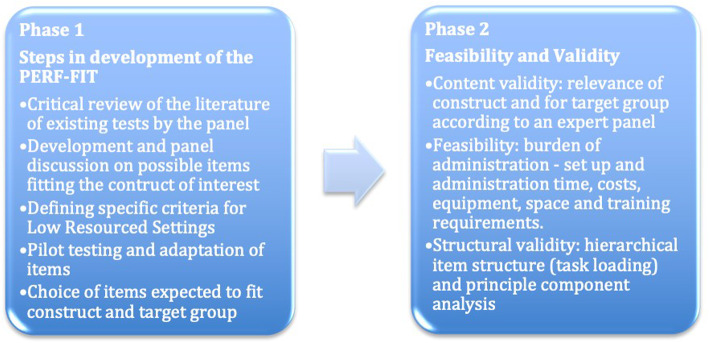
Steps in the development and validation process of the PERF-FIT

This section will be presented in two phases (See Fig. 1). Phase one will provide a description of the PERF-FIT and explain the steps taken to develop the test items. In the second phase, we will provide preliminary evidence to support the validity and feasibility of the test.

### Phase 1: test description and development

#### Description of PERF-FIT

The PERF-FIT was designed as an instructor-administered, cross-culturally comparable, functional measure of skill-related physical fitness for children aged 5–12 years. The main rationale was to develop a low-cost, and easy-to-administer measure that could be used across a variety of low-income contexts. The PERF-FIT focuses on skill-related physical fitness and is divided into two subcomponents; *motor performance,* and *agility and power* subscales.

##### Motor performance *subscale*

The motor performance component or the *skills item series (SIS*) consists of five items including jumping, hopping (left and right), bouncing and catching, throwing and catching, and balance. These tasks are administered to the child in an increasing order of difficulty (task loading). The child starts at the easiest level and ends at the most difficult level within the same task series. The items within a particular task/skill series are terminated when the child is unable to achieve the minimum points after two consecutive trials. No second trial is performed when the maximum points are attained. For example, in the jumping task series, distance and height are modified to increase the difficulty level of the task after the initial trial. Each child is given a practice trial before performing the test trials. To avoid fatigue 15 s rest is allowed between trials. The child’s performance during the test trials are recorded and used for calculating the item score (See additional file 1 for items and scoring).

##### Agility and power *subscale*

The agility and anaerobic power component has five items. These are running, stepping, side-jump, long jump, and overhead throw. All items are initially demonstrated before the child is asked to perform one practice trial. Following this, each child is expected to perform two test trials, with 15 s rest interval. The child’s performance during the test trials is recorded and best scores are used in the analysis.

#### Developmental process

The development of the PERF-FIT involved a three-step process and included (1) development of a conceptual framework (2) selection of initial item set and expert evaluation and (3) pilot testing.

##### Conceptual framework

A literature search was first conducted to define the domains of skill-related physical fitness that were thought to be important for children’s development and growth. Based on our search, six key skill-related physical fitness attributes were identified and defined. These included agility, power, balance, coordination, speed and reaction time (Table 1). The outcomes of the chosen items are product-oriented in order to test the quantitative level of performance in tasks that are considered to be basic skills of children at elementary school age.

**Table 1:** Theoretical and operational definitions of skill-related physical fitness (adapted from [14])

Domain	Theoretical definitions	Operational definitions
Agility	The ability to rapidly change the position of the entire body in space, with speed and accuracy.	The child is asked to jump side to side at high speed and with great efficiency.
Balance	The maintenance of equilibrium while stationary or in motion.	The child is asked to stand on one leg while holding on to his free foot for an extended period of time (static). The child is asked to grasp his/her unsupported foot while making slow steps in the agility ladder (dynamic) The child is asked to move his arms and trunk forward to pick up an empty can while standing on one leg.
Coordination	The ability to use the senses, such as sight and hearing, together with moving body parts, in performing motor tasks smoothly and accurately.	The child is asked to time arm and leg movements to perform a catch or make consecutive jumps smoothly.
Power	The rate at which a person can perform work (strength over time).	The child is asked to apply great force to a heavy object to make a throw or propel the whole body forwards to make a long jump.
Speed	The ability to perform a movement within a short period of time.	Running speed is tested in the agility ladder where the child is moving forward from the start line quickly to the end of the ladder, turn and run back.
Reaction time	The time elapsed between a stimulus and the beginning of the reaction to it.	Most items have a start signal to which the child has to respond; many adaptation made to make a movements successful are based on rapidly responding to a stimulus and adapt the movement to that stimulus to avoid stepping on a bar (agility items) or estimate the trajectory of a bouncing or thrown ball.

These domains were selected because of their cross-cultural applicability, relevance to childhood routines and games, ease of testing in low-income schools and their ability to enhance physical activity across the lifespan. In addition, we wanted to ensure that the relationship between (anaerobic) fitness and motor skills is acknowledged in diverse low-income communities [24] and to create more awareness that evaluation of skill-performance related physical fitness has important implications for child development, active lifestyle, physical education and policy development [25].

##### Item identification

Based on the literature search and pre-defined criteria (see Table 2), 20 items representing the six core domains of the theoretical framework were identified and reviewed by a panel of experts (all experienced clinicians with doctorate degrees; three from Africa, two from South America, and two from Europe). Based on the experts’ feedback, 10 items were finally included in the PERF-FIT test battery. The 10 items were divided into two subscales as mentioned earlier (i.e. motor performance and agility and power components). Since most items have a start signal to which the child has to respond no separate reaction time item was included. The theoretical and operational definitions of each domain were clarified to guide task identification (See Table 1). Further, the process of increasing the difficulty of tasks (task loading) for each item was defined to facilitate implementation and interpretation of scores. (See Additional file 2 for the items and the increase in difficulty).

**Table 2:** Criteria for selecting items of the PERF-FIT test battery

Criteria	Description
A	Tasks should be able to measure skill-related physical fitness.
B	Tasks should allow for progressive increase in difficulty (i.e. task loading).
C	Tasks should have cross-cultural applicability and be appropriate for children aged 5–12 years
D	There should be no specific space restrictions for testing
E	Materials needed for the test should be affordable to people working in low-income settings. (See Additional file 3)

##### Pilot testing

The last stage of the development of the PERF-FIT was the pilot-testing phase. The final items were tested in two small samples involving South African children (*n* = 10 and *n* = 20) to assess feasibility, acceptability, ease of administration and implementation challenges. Feedbacks provided by the children and test administrators were used to refine aspects of the test items, and to improve the clarity of the instructions and scoring forms. These were done to reduce the burden of administration (for an example of an item description see Additional file 2). Instructions for obtaining the standardized materials were also provided in the manual (see Additional file 3).

### Phase 2: validation

#### Content validity

Seven experts (i.e. four pediatric physical therapists, one occupational therapist, and two physical educators) with several years of experience in movement skills and fitness assessments were asked to assess the relevance of the PERF-FIT items. Each expert had experience in test development and had worked with children in low-resource settings for more than 10 years. According to the COnsensus-based Standards for the selection of health status Measurement INstruments (COSMIN) guidelines a moderate sample size (5–9 experts) is rated as good to assess if all items are relevant for the study population (content validity) [26]. Experts were required to indicate whether the PERF-FIT items reflected the constructs they were intended to measure. In addition, they were asked to evaluate the appropriateness of the selected tasks for the target population for the intended future use of the test (typically developing children and children with poor motor coordination, e.g. Developmental Coordination Disorder (DCD), Attention Deficit Hyperactivity Disorder (ADHD), or Fetal Alcohol Syndrome (FAS) Learning Disabilities (LD), in low-resourced communities). This evaluation was done using a 4-point scale developed based on the criteria proposed by Davis [27] (Score *1 = not relevant, 2 = somewhat relevant, 3 = quite relevant, 4 = highly relevant)*. The Content Validity Index (CVI) was used as an estimate of the content validity of each variable in the test battery. Additionally, Scale-level Content Validity Index (S-CVI), representing the overall content validity of the PERF-FIT, was computed as the average of the I-CVIs for all the test items [28].

#### Feasibility

The feasibility of the test was assessed by looking at 1) acceptability (based on participants and assessors’ perspectives), 2) adverse events or injuries 3) burden of administration - set up and administration time, cost, equipment, space and training requirements. The experiences of the assessors were also captured by self-report.

#### Structural validity

The PERF-FIT test was validated in a convenience sample of 80 Brazilian children aged 7–12 years (mean 9.2 SD 1.1). Following the COSMIN guideline in sample size 80 children was deemed acceptable for this kind of study [26]. Participants (39 boys; 41 girls) were recruited from two primary schools in a low-socioeconomic area in the state of Sao Paulo. Children were excluded if they had any injury or physical disability that hindered their involvement in the assessments. Written informed consent was obtained from parents and each child provided assent before involvement. Ethical approval was obtained from the Human Research Ethics Committee of the Federal University of Sao Carlos (89,993,118.8.000.5504/2018). Permission was also obtained from the head teachers of the schools. The PERF-FIT was administered by a team of trained assessors. The assessors received a six-hour training on test administration, which was facilitated by the lead author. The training consisted of lectures and practical demonstrations. After the training, the assessors practiced instructions and test items in small groups to obtain a solid grasp of the tasks and scoring scheme. The assessors were pediatric physiotherapists, occupational therapist, and a physical educator who had general knowledge of strength and conditioning principles, and typical child development. Each assessor received a test manual for reference. Testing took place in the school premises and children were assessed in pairs. However, in situations where children were too distracted or absent from school, they were tested individually on a separate day. Data collection was completed within a space of 3 weeks. Structural validity was evaluated by testing the linear increase of the loading used to make the items more difficult (see Additional file 1) and by exploratory factor analysis on the data of the 80 children.

### Statistical analysis

Descriptive statistics such as means, standard deviation, and frequencies were used to summarize the data and experts’ responses. The Content Validity Index (CVI) was used as an estimate of the content validity of each variable in the test battery. CVI is the most widely used quantitative approach for the content validation of instruments. Specifically, Item Content Validity Index (I-CVI) was computed for each test item as the number of experts giving a rating of either 3 (quite relevant) or 4 (highly relevant), divided by the total number of experts in the study. Scale level-Content Validity Index (S-CVI), representing the overall content validity of the PERF-FIT, was computed as the average of the I-CVIs for all the test items. The adopted cut-off for an acceptable level of I-CVI was > 0.78 and S-CVI of greater than 0.90 qualifies the test battery for excellent content validity [28].

Further, structural validity of the SIS was checked by examining the hierarchical sequence of the items visually, and by repeated measure ANOVA. In order to test for linearity, maximum scores for the easier items of jumping, hopping and balance were divided by 2 to make the maximum score for all items equal (i.e. 4 points). Structural validity was also examined by exploratory factor analysis. A principal component analysis (PCA) with Varimax rotation with Kaiser-Meyer-Olkin (KMO) test was performed. Eigenvalues greater than one were used to determine the number of dimensions in the PERF-FIT. Data analyses were performed using SPSS (version 24.0, SPSS Inc., Chicago, IL, USA).

## Results

### Content validity

The median score for all the questions on the relevance of constructs and items for target group was four. All the experts deemed the five SIS “quite relevant” or “highly relevant” in measuring motor skills performance. The same trend was evident for agility and power items. One therapist scored a 2 for “throwing and catching” because she had preference for manual dexterity items instead of throwing and catching.

The experts were unanimous in their responses regarding the relevance of the items for the target population. Five deemed the items “highly relevant” in measuring the constructs of interest in children in low-income settings. Two experts chose the “quite relevant” more often than “highly relevant”.

I-CVI for the Throw and Catch item was 0.86 and 1.00 for the other nine items, leading to a S-CVI of 0.99, indicating excellent content validity.

### Feasibility

#### Acceptability

Acceptability of the PERF-FIT test was high, both the participants and assessors liked the test items. Participants demonstrated great understanding regarding how the task difficulty was increased in the SIS, which was consistent for jumping and hopping items. If a participant could not make the jumps or hops over the foams, they jumped on them or scattered them and in most cases they helped with rearranging the materials for the next trial. Additionally, most participants were not comfortable with the stork balance item. This was because touching their stance leg with the other foot sometimes made their trousers dirty. Twenty-four percent (24%) of the participants performed the test without shoes because they lacked the appropriate footwear for running or jumping. In 41% of the items of the skill item series, participants obtained the maximum score during the first trial on the given task. However, 11% of the participants were still able to obtain the maximum scores during the second trial (ranging from 3% extra maximum scores on the left to 18% on the right foot for the balance item; for the jump and hop items, the percentages varied from 9 to 14%). Asking participants to re-take an item in cases when the maximum score was not obtained was found to be acceptable for the majority of the participants. However, in situations when a child scored just enough points on an item after the three trials (one practice- and two test trials) in a row on two difficult levels, the hopping items were reported to be quite tiring, although the children alternated the right and left legs and were allowed 15 s rest interval.

#### Adverse effects or injuries

As previously indicated, the participants were able to complete testing procedures without reporting injuries. Also, no severe adverse events or complications were observed by the assessors. Only one participant out of the 80 fell during testing, and some participants complained of tiredness if they had to execute two trials (first and second trial) but no injuries were observed. All but one child was able to perform all the test items. That child complained of pain in his right knee during the hopping tasks. Two children needed extra recovery time because they ran out of breath after completing the agility tasks. One girl had severe fear of failure and needed extra encouragement to finish the test. Another girl had strong reading glasses and needed to take them off because moving made her “dizzy”. Though children showed some short-lasting signs of tiredness, which fits into the intended construct to be measured, a greater proportion of participants completed the tasks without getting exhausted.

#### Burden of administration

It was observed that the time needed to complete the test depended on the participant’s skill level. Overall, it took approximately 20–40 min to complete the full test per child. If the child is only able to do the first level of the SIS, less than 20 min was needed. If two children were tested at the same time, it took about 30–50 min to complete all items because items only had to be demonstrated once and children could alternate so they had the required rest between items. Also, it took about 10 min for the assessors to set up and to pack the test materials. The test forms were easy to fill out, not many disputable situations occurred, as reported by the assessors. Mistakes made by the children were usually easy to identify (e.g., missing a catch, losing balance, jumping on the foams, or stepping on the bars of the ladder). The equipment was found to be relatively cheap (less than 50 US dollars). Just a firm surface was needed for testing and the dimensions were 5–6 m long by 3–4 m wide. Assessors were encouraged to walk along the agility ladder with the child to be able to check if the child stepped on the bars whenever the foams were used. The pieces of foam were found to be bulky (though light) for transportation to the different schools.

### Structural validity

**Fig. 2:**
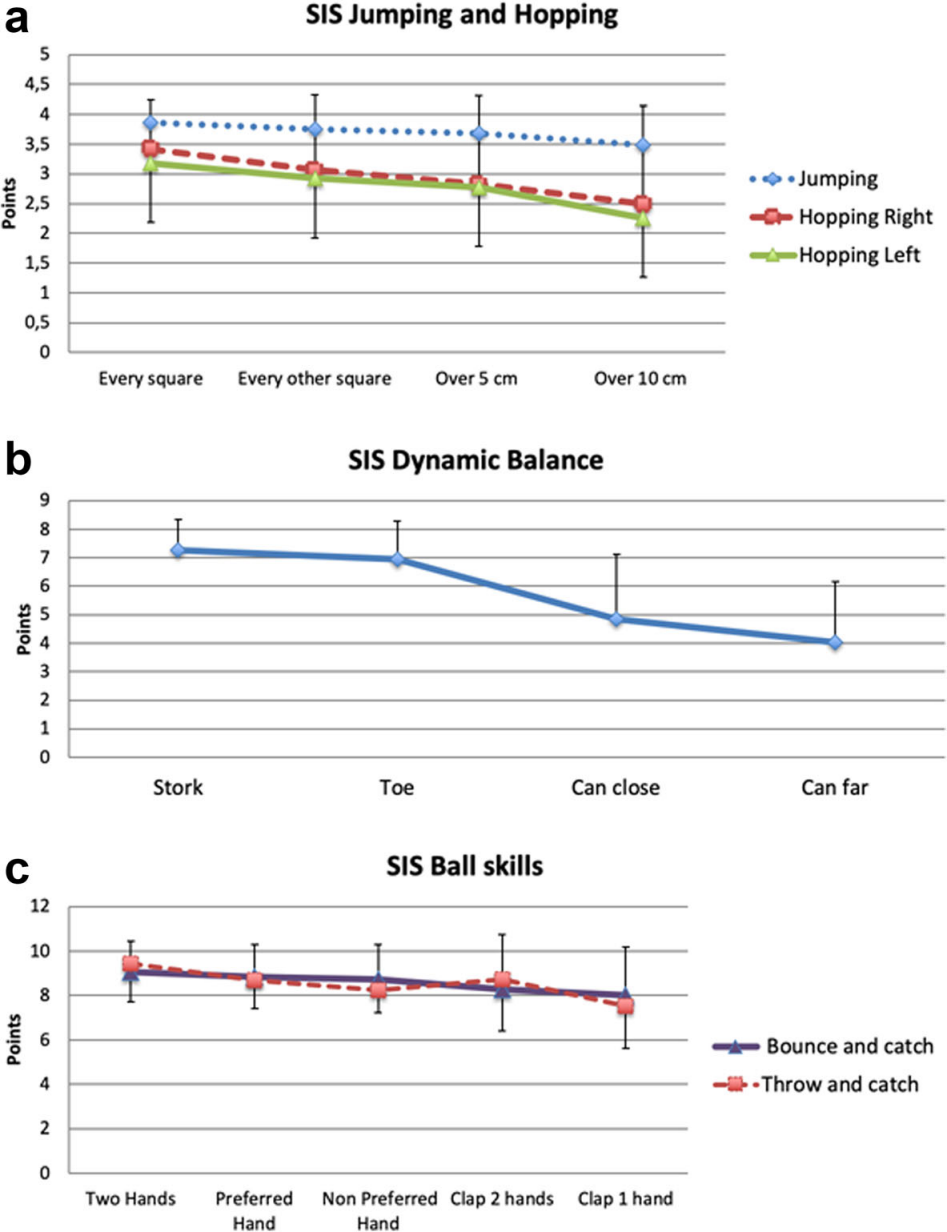
**a**, **b** and **c**. Mean scores on the Skill Item Series; **a**) Jumping and Hopping, **b**) Balance and **c**) Ball Skills. Error bars depict standard deviations

Hierarchical sequence of the items was depicted by plotting the mean score over the same tasks with increasing difficulty. Figure 2 a, b and c show that scores decrease with increasing difficulty. For the entire SIS (jumping, hopping, bouncing and dynamic balance) the repeated measures ANOVA confirmed this decrease in scores was highly significant (*p* < 0.001) and linear. It was only in the case of throwing that the mean for catching after a clap with two hands was found to be easier than to catch with the non-preferred hand without a clap. As depicted in 2b it was clear that the increase between the two dynamic balance tasks and the “Can” tasks was large which was confirmed by a higher order polynomial effect (*p* < 0.001).

A principal component analysis was performed with 13 item variables (see Table 3) and Varimax rotation. The KMO test showed that the sample size was adequate for performing a PCA (KMO = 0.85) as recommended by Hutcheson and Sofroniou [29]. Bartlett’s test of sphericity was significant, showing a sufficiently high correlation between the variables for a PCA. Three components had eigenvalues above the Kaiser’s criterion (Eigenvalue of greater than 1) and could together explain 63.5% of the variance. The factor loads on the components suggest that the first component is represented by “Locomotor skills that require static and dynamic balance”, the second component by “Throwing and catching” and the third component by “Agility and power “ (see Table 3). Throwing the heavy bag loaded with the other ball skills not with the agility and power. The normal running in the agility ladder loaded on both Locomotor and Agility factor.

**Table 3:** Factor analysis of the PERF-FIT items, values over 0.40 are shown in the columns

Rotated Component Matrix			
	Component		
	Locomotor/Balance	Object control/Ball skills	Agility/power
1. Jump (#)	.746		
2. Hop Right (#)	.770		
3. Hop Left (#)	.721		
4. Static Balance Right (s)	.741		
5. Static Balance Left	.528		
6. Dynamic Balance (#)	.631	.458	
7. Bounce and Catch (#)		.797	
8. Throw and Catch (#)		.711	
9. Overhand throw (cm)		.762	
10. Long jump (cm)		.410	.434
11. Running (s)	−.533		−.493
12. Stepping (s)			−.796
13. .Side Jump (#)			.851

Extraction Method: Principal Component Analysis

Rotation Method: Varimax with Kaiser Normalization

#; number of times; s: second; cm: centimeter

## Discussion

The purpose of this study was to describe the development, feasibility and validation (content and structural validity) of the PERF-FIT test battery. Our hope is to use this test battery to gather data on motor skills and physical fitness at the population level among children living in low-resource communities, where these assessments have historically received little attention. Overall, the PERF-FIT was found to be feasible and implementable in a low-resource context. Additionally, the test was deemed to have excellent content and good structural validity. Structural validity was confirmed with exploratory factor analysis revealing three factors. The PERF-FIT test battery does not only assess different motor skills (for instance locomotion combined with stability) but also evaluates motor coordination and anaerobic power, which are embedded in different skill sets (for instance hopping 4 times sequentially over foams with a height of 10 cm or throwing a 2 kg sandbag). Since the PERF-FIT is intended to measure a combination of skills and muscular fitness, the way it is structured appears to be more appropriate than testing coordination and power in isolation. The three factors (locomotor skills and balance, throwing and catching, and agility and power) that emerged in the factor analysis confirmed that the PERF-FIT is suited for measuring three major aspects of motor behavior in children. These factors seem to match the definition of fundamental motor skills [29], which includes locomotion (e.g. running and hopping), manipulative or object control (e.g. catching and throwing) and stability (e.g. balancing and twisting) skills [30, 31]. An unexpected finding was that the explosive power item (long jump) and dynamic balance clustered with the object control items (balls and heavy bag) albeit with lower loading. Future studies may have to confirm the common denominator for these skills.

Evidence-based intervention approaches for children with poor motor skills such as task-oriented training tend to focus on the meaningful tasks, and cultural and contextual demands of a given task [32]. Since we intended to develop a low-cost test, we tried to use task-based, gross motor skills, which could be easily reproduced. We also measured different levels of the same task (Skill Item Series). We also added components of anaerobic power and agility to make the test comprehensive enough to assess relevant motor skills and anaerobic fitness variables required for participation in daily activities. Another reason was to provide a test that will align with the new guidelines on assessment and intervention for children with DCD [32]. Failure of anaerobic power and agility may have detrimental effects on children’s participation at the playground, home and in their communities.

Our PERF-FIT test has comparable features to two previous tests, which were developed in Canada. The first is called the Canadian Agility and Movement Assessment (CAMSA) [21], a test that measures fundamental, complex and combined movement skills. This test is a combination of movement skills and muscular fitness items, an approach which is similar to that used in the development of the PERF-FIT test battery. The CAMSA requires the child to complete seven different movement skills as fast as possible after each other in a circuit set up in a gymnasium. The movement skills included are: (1) 2-footed jumping into and out of 3 hoops on the ground, (2) sliding from side to side over a 3 m distance, (3) catching a ball and then (4) throwing the ball at a wall target 5 m away, (5) skipping for 5 m, (6) 1-footed hopping in and out of 6 hoops on the ground, and (7) kicking a soccer ball between 2 cones placed 5 m away [21]. The CAMSA is set up in a circuit fashion which makes it very useful when larger groups need to be tested on a combination of skills and fitness in a relatively short time. Another interesting feature of the CAMSA is that it combines process and product outcomes. One point is awarded for each skill criterion performed correctly (process) and an overall time for the whole circuit is used (product). Both the time score to complete the CAMSA (range 1 to 14) and the criterion-referenced assessment of skill performance (range 0 to 14) are combined in a Total score. The CAMSA is a good option to follow large groups of children over time in a physical education context and gives a good broad index of the children’s performance [21]. Because it uses process criteria it requires administrators with good observational technique. It is harder to use this tool in clinical testing of one child or for pre-post intervention comparison because it combined so many factors in one total score. The CAMSA is suitable for children aged 8–12 years, which partially overlaps with age range for the PERF-FIT.

Similarly, the PERF-FIT test shares certain characteristics with the University of Québec in Chicoutimi and University of Québec in Montréal (UQAC–UQAM) test. The UQAC–UQAM is a multi-skill test designed for children between 6.0 and 12.99 years. The test contains 11 items [22, 23] including four agility items: 5x5m shuttle run; sidestep run; slalom run; circle run; four coordination items: eyes/hand coordination (ball toss); hand/foot coordination; balance eyes open (on the beam); balance eyes closed (on the ground); and three reaction time or speed items: simple reaction time (on a computer); lower limbs speed (2 foot tapping); upper limb speed (plate tapping). Clearly this test focuses more on fitness. Further, the UQAC–UQAM has specific items not included in the PERF-FIT (like upper and lower limb tapping). However, it contains fewer items measuring fundamental motor skills, like catching, jumping or hopping. For instance, for the highest level of the catching and throwing items series of the PERF-FIT children can reach a total of 50 balls caught in 5 steps of difficulty (10 per level). In comparison, the target ball toss test used in UQAC–UQAM measured the ability to execute a ballistic movement with the dominant hand using an overhand precision throw (10 times). Points are giving depending where the ball hits the bull’s eye form a 5 m distance [22, 23]. This is definitely a fun game-like test item for children but in our context finding an even wall for the target and the large space (> 7 m) needed for UQAC–UQAM would have been problematic.

The PERF-FIT has cross-cultural applicability and can be used in diverse resource-limited environments. The test battery could be administered outside when the weather is good or inside on a rainy day. The acceptability of the test seems to be good both for the children and the assessors. Because some assessors experienced challenges demonstrating the more difficult items, we have developed instructional video clips that can be used to train to assessors and can be shown on a phone or as pictures to the children. However, it was clear that after children were shown the easiest level of the SIS, the harder items did not have to be demonstrated because the children implicitly knew the next level of the task.

This study revealed that some minor adaptations are needed. Foam blocks will be made smaller without changing the task difficulty. We will use half the width in our next validation study. The order of the ball items was not changed, because we want to keep the order in bouncing and catch and throwing and catch the same to avoid errors in administration. Because children complained of dirt on their trousers after doing the stork balance task, we will add the standing knee pose (comparable to the standing knee pose on the Wii balance board; to replace the stork balance task) [33, 34]. To avoid too much fatigue, we will increase the number of points that children need to attain before proceeding to the next difficultly level. We therefore intend to increase the number of points that children need to obtain to go to the next level from 50% to greater than 50%. After these adaptations we have started collecting normative data in areas for which the test is meant to be used.

### Strength and limitations of the study

In accordance with the COSMIN guidelines [26], we sampled data from a group of children in one of the targeted low-resource areas and involved the potential users (researchers and clinicians) in the development and evaluation of the test items on relevance and suitability for the target population. Pilot testing was conducted and a relatively large sample was tested to evaluate feasibility and aspects of validity in a typical low-resourced community. Assessors reported the test was easy to administer and the time required to administer the tests was reasonable (i.e. maximum 40 min per child). We had no safety issues, adverse effects or injuries.

A limitation of the study is that the data were derived from only two schools and testing was performed during the hot season, which may have influenced children’s performance. This limits generalizability of our findings. Moreover, no 6-year-old child participated in this initial study. Younger children are more likely to be distracted and may not understand the test items as easily as the seven-year olds. Lastly, the results need to be confirmed in diverse cultural contexts in Africa and South America as well as in specific populations including children with DCD, ADHD, FAS, underweight, obesity or learning disabilities.

## Conclusion

The PERF-FIT test battery seems to be a feasible assessment that can be implemented in low-resourced communities. The test is viewed to have relevant items and possesses excellent content and good structural validity. People working with children could use this test to measure children’s skill-related physical fitness. After minor adaptions the PERF-FIT test battery is ready to gather normative values on skill-related physical fitness in young children in low-income settings. More research is needed to evaluate its reliability as well as criterion and cross-cultural validity.

## Supplementary information

**Additional file 1.**

**Additional file 2.**

**Additional file 3.**

## Data Availability

The datasets used and analysed during the current study are available from the corresponding author on reasonable request. The PERF-FIT manual and instruction videos can be accessed free of charge for the intended users after registration via the first author for use in low resource communities.

## Abbreviations

ADHD: Attention Deficit Hyperactivity Disorder
BOT-2: Bruininks-Oseretsky Test of Motor Proficiency- second edition (BOT-2)
CAMSA: Canadian Agility and Movement Skill Assessment
COSMIN: COnsensus-based Standards for the selection of health status Measurement INstruments guidelines
CVI: Content Validity Index
DCD: Developmental Coordination Disorder
FAS: Fetal Alcohol Syndrome
I-CVI: Item Content Validity Index
KMO: Kaiser-Meyer-Olkin test
KTK: Körperkoordinationstest für Kinder
LD: Learning Disabilities
MABC-2: Movement Assessment Battery for children, second edition (MABC-2]
PCA: Principal Component Analysis
PERF-FIT: Performance and Fitness test battery
S-CVI: Scale-level Content Validity Index
SIS: Skills Item Series
UQAC: University of Québec in Chicoutimi
UQAM: University of Québec in Montréal
