# Supplementary material for: Feasibility and content validity of the PERF-FIT test battery to assess movement skills, agility and power among children in low-resource settings
Source: BMC Public Health. 2020 Jul 20;20:1139. doi: 10.1186/s12889-020-09236-w (PMC7372755; doi:10.1186/s12889-020-09236-w)
Supplement: Supplementary file 1 — Additional file 1. [file 12889_2020_9236_MOESM1_ESM.docx]

Additional file 1: The PERF-FIT items

Subtest Agility and Power

Running in agility ladder 2 trials (Time in 0.01 s and number of mistakes)

Stepping in agility ladder 2 trials (Time in 0.01 s and number of mistakes)

Side jumps 2 trials (Number of correct jumps in 15 seconds)

Long Jump 2 trials (Distance in cm)

Throwing for distance: sandbag 2 trials (Distance in cm)

Subtest Motor Skill Performance

Bounce and catch 10 trials: 5 items 10 point=50 points max score.

1. Bounce the ball to the floor and catch it with two hands
2. Bounce the ball on the floor and catch with the preferred hand
3. Bounce the ball on the floor and catch with the non-preferred hand
4. Bounce on the floor with the preferred hand, clap and catch with the preferred hand.
5. Bounce on the floor with the non-preferred hand, clap and catch with the non-preferred hand.

Throw and catch 10 trials: 5 items 10 point=50 points max score.

1. Throw the ball in the air and catch it with two hands
2. Throw the ball in the air and catch with the preferred hand
3. Throw the ball in the air and catch with the non-preferred hand
4. Throw the ball in the air, clap and catch with the preferred hand
5. Throw the ball in the air with the non-preferred hand, clap and catch with the non-preferred hand.

Static balance: 2 items for each leg 15s; 2 legs x 2 items x15s= 60 seconds max time score;

1. Balance Hug Knee Static R and L (timed max 15s per leg),
2. Balance Grasp Foot Static R and L (timed max 15s per leg).

Dynamic balance: 4 items x 8 points =32 max balance score.

1. Balance Hug Knee Dynamic (count 8)
2. Balance Grasp Foot Dynamic (count 8)
3. Balance Pick up Cans Dynamic R and L (count 8: per leg).

Jump: 4 items 8, 4, 4, 4 points=20 points max score.

1. Jumping in each square (8 jumps)
2. Jumping every other square (4 jumps)
3. Jumping over four 5 cm foams (4 jumps)
4. Jumping over four 10 cm foams (4 jumps).

Hop: 4 items for each leg 8, 4, 4, 4 points=20 points per leg 40 points max score.

1. Hopping each square (8 hops)
2. Hopping every other square (4 hops)
3. Hopping over four 5 cm foams (4 hops)
4. Hopping over four 10 cm foams (4 hops).

Fatigue and rest

In order to alternate between more and less fatiguing items the following order of the items and rest periods for the full test procedure are recommended:

- Two slow practice trials of the agility ladder are given as general warming-up for the test
- Running (two slow practice trials of the agility ladder are given as general warming-up for the test, give a minimum 15s rest between the 2 test trials)
- Stepping (minimum 15s rest between the 2 test trials)
- Side jump (minimum 15s between the 2 test trials)
- Long jump (minimum 15s between the 2 test trials)
- Throw sandbag (minimum 15s between the 2 test trials)
- Series Bounce and Catch (no rest needed)
- Series Throw and Catch (no rest needed)
- Series Hug knee (Preferred leg first, no rest needed) Static and Dynamic
- Series Grasp toe (Preferred leg first, no rest needed) Static and Dynamic
- Series Pick up cans (Preferred leg first, no rest needed)
- Series Jumping (4 items) (minimum 15s between the 4 items)
- Series Hopping (4 items) (alternate right and left leg and minimum 15s between the items performed on the same leg).
