# Supplementary material for: Feasibility and content validity of the PERF-FIT test battery to assess movement skills, agility and power among children in low-resource settings
Source: BMC Public Health. 2020 Jul 20;20:1139. doi: 10.1186/s12889-020-09236-w (PMC7372755; doi:10.1186/s12889-020-09236-w)
Supplement: Supplementary file 2 — Additional file 2. [file 12889_2020_9236_MOESM2_ESM.pdf]

Additional file 2: Example of an item description and scoring

Item 3. Side jump

Equipment

- The first 3 squares of the Agility ladder
- Timer

Task

Jump sideways on 2 feet. One foot per square, jump sideways in the same 3 squares of the agility ladder (see photos).

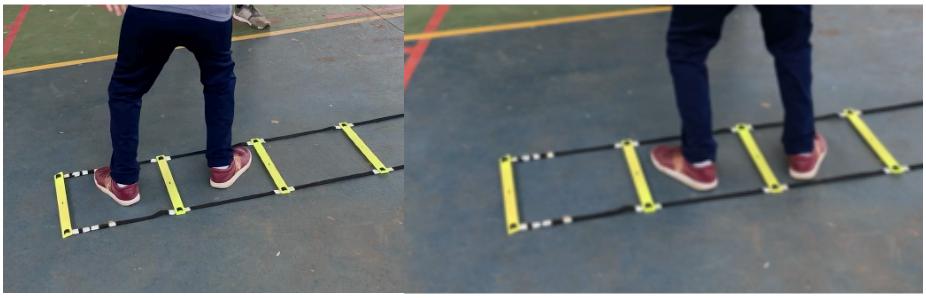

General instructions

"What I want you to do now is jump on both feet from these squares in the other ones, sideways. (Show the child how to jump, start slowly, then you may go faster).
Try to jump as often as you can in 15 seconds. I will tell you when to stop.
"Try it a few times (5 jumps), save your strength".

If the child doesn't get the task show again and give extra practice (one time an extra 5 jumps). After practice we will do the real test twice. "You start after I say ready steady GO, got that? "

Always give 2 test trials. Rest time between the 2 trials is 15seconds. Tell the child *to keep going* if it makes a mistake (but don't count the jumps with a mistake, see scoring).

Scoring

Count the number of correct landings (=both feet in the correct squares) in 15 seconds while not stepping on the yellow bars.
If the child falls (totally steps out of the agility ladder, or falls on the floor), only count the correct jumps before the loss of balance.
If the child can restore balance **without being out of the ladder with 2 feet** you can continue counting the correct jumps.
