# Supplementary material for: Feasibility and content validity of the PERF-FIT test battery to assess movement skills, agility and power among children in low-resource settings
Source: BMC Public Health. 2020 Jul 20;20:1139. doi: 10.1186/s12889-020-09236-w (PMC7372755; doi:10.1186/s12889-020-09236-w)
Supplement: Supplementary file 3 — Additional file 3. [file 12889_2020_9236_MOESM3_ESM.docx]

## Additional file 3

## Materials needed for the PERF-FIT

1 agility ladder with nine (35x35 cm) squares and 3 cm wide rungs (see schedule)

4 sheets of paper with a red cross

1.5 liter pet bottle filled with water

8 pieces of foam (40 x 20 cm wide and 5 cm high)

4 soda cans (weight 13 oz or 0.368 kg)

2 kg sandbag (20x20 cm)

Tennis ball

Colored string or lace or sticker to indicate left and right for young children

Tape measure

Stopwatch


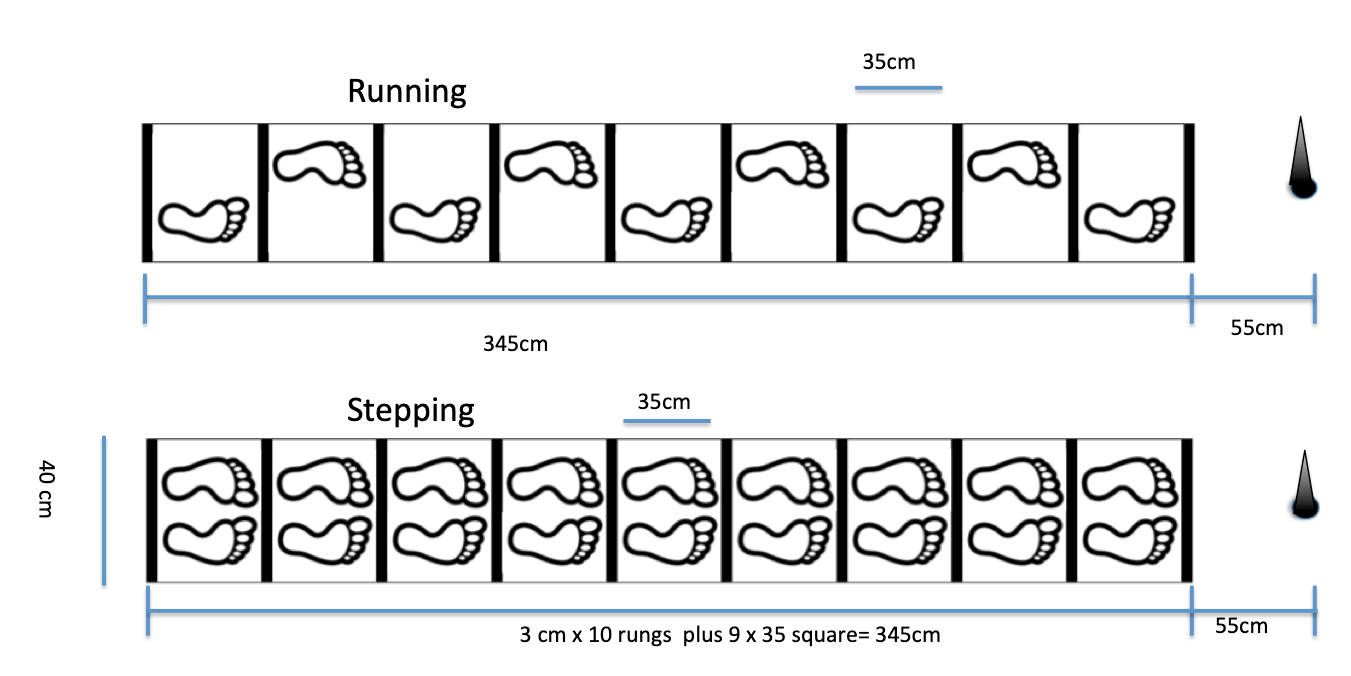


The PERF-FIT manual and the instruction videos to learn the test can be accessed free of charge after registration via the first author for use in low resource communities. [bouwiensmits@hotmail.com](mailto:bouwiensmits@hotmail.com)
